# Odontogenic Fibroma of the Maxilla - An Atypical Presentation

**Published:** 2016-12-24

**Authors:** Arpita Kabiraj, Anil Singh, Tanya Khaitan, Amrita Jaiswal

**Affiliations:** 1 *Dept. of Oral Pathology & Microbiology, Index Institute of Dental Sciences, Indore, Madhya Pradesh, India*; 2 *Dept. of Oral Pathology & Microbiology, Saraswati Dental College, Lucknow, U.P, India*; 3 *Dept. of Dentistry, Murshidabad Medical College and Hospital, Berhampore, West Bengal, India *; 4 *Dept. of Prosthodontics, Sardar Patel Post Graduate Institute of Dental & Medical Sciences, Lucknow, U.P, India*

**Keywords:** Mesenchyme, Neoplasm, Odontogenic Fibroma, Odontogenic Epithelium

## Abstract

Odontogenic fibroma (OF) is considered an unusual, benign mesenchymal neoplasm and one of the most little-understood lesions amongst all odontogenic neoplasms. The incidence rate of the tumor is from 0% to 5.5%. WHO classified them into intraosseous or central and extraosseous or peripheral variants. It chiefly consists of fibroblastic tissue with an inconsistent amount of inactive appearing odontogenic epithelium. The lesion has a slow growth along with cortical expansion with equal predilection in the anterior maxilla and posterior mandible. Radiologically, multilocular radiolucency is the most frequent finding with few cases being associated with root resorption or displacement. Microscopically, mature collagen fibers and numerous fibroblasts along with odontogenic epithelial islands are characteristically found. Central Odontogenic Fibroma responds well to surgical enucleation with no tendency for malignancy or recurrence. Here we report a rare case report of an 18 yr old male patient with Odontogenic fibroma of the posterior maxilla.

## Introduction

The Odontogenic fibroma (OF) is an unusual, atypical, benign tumour with defined margins and of mesodermal origin (1). It is an obscure and controversial tumour: obscure due to its rare nature (it represents 0% to 5.5% of all odontogenic tumours recorded in studies exploring a minimum of 300 odontogenic tumours) and controversial because of the improbability as to the number of different types elucidated (2, 3). The WHO classified OF as a connective tissue tumour consists of collagenous bundles and fibroblasts with an uneven amount of apparently inactive odontogenic epithelium inactive. It has been classified into two cases: intraosseous or central and extraosseous or peripheral (4, 5).

Central odontogenic fibroma (COF) is benign and distinctive variant, small or extensive, probably has finite growth. Extensive bone destruction and expansion of bone indicate the aggressive nature of the lesion. Thus, not many cases of this unusual lesion have been recorded and published. According to a literature, only 7 cases have been reported in the last 5 to 6 yr tenure. 

This paper reports a case of odontogenic fibroma in the posterior maxillary region of an 18 yr old male patient.

## Case report

An 18 yr old male patient presented with pain in the left upper posterior region of the jaw since 2 months. The pain was dull and intermittent in nature that aggravated on mastication or pressure and relieved on taking medication. It was occasionally associated with pus discharge. Past dental history revealed that he had undergone extraction of upper left posterior tooth due to caries. The patient also reported with good systemic health. 

There were no conflicts of interest and no financial issues regarding this case.

**Fig. 1:**
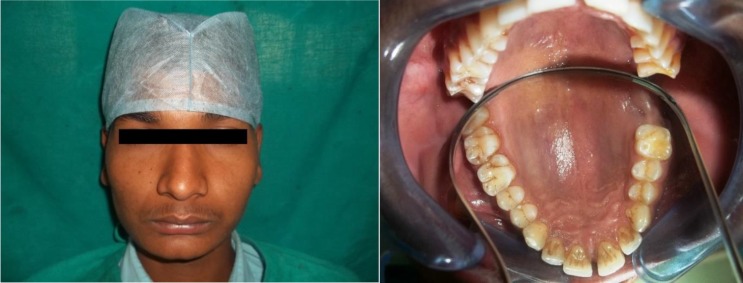
Extraoral photograph showing no facial asymmetry (a); intraoral photograph showing no significant swelling or vestibular changes (b

There was no facial asymmetry seen extra orally. On intraoral examination, no significant swelling or vestibular changes were evident. Clinically, 27 and 28 were missing. [Fig. 1 a,b] The alveolar bone over that region appeared normal and was tender on palpation

**Fig. 2:**
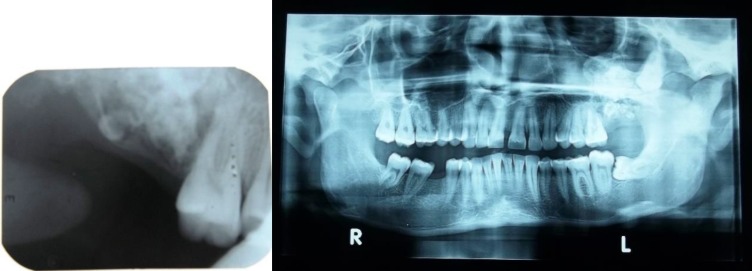
IOPA showing irregular radiopaque masses (a); OPG showing well-defined radiolucency present in the 27, 28 region (b

**Fig. 3:**
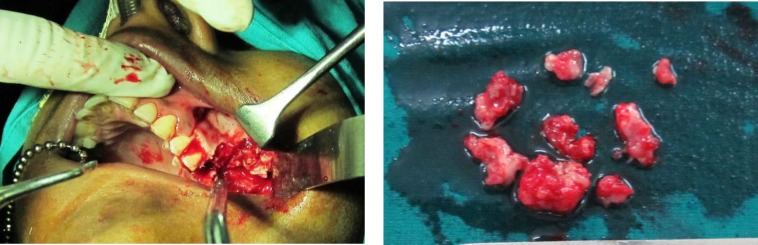
Photograph showing surgical intervention and the gross specimen after surgery (a & b

The intraoral periapical radiograph showed irregular radiopaque masses distal to 26 in the alveolar bone. OPG showed a well-defined radiolucency present in the 27, 28 regions that extended from distal of 26 to maxillary tuberosity region surrounded by a white sclerotic border with intermittent irregular radiopaque masses within. Vertically impacted 28 were seen embedded in the left zygoma region. [Fig. 2 a, b] based on the clinical and radiological findings, a provisional diagnosis of an odontogenic tumour was wellthought of. Surgical intervention was performed and the tissue specimen was subjected to histopathological assessment [Fig. 3 a, b].

**Fig. 4:**
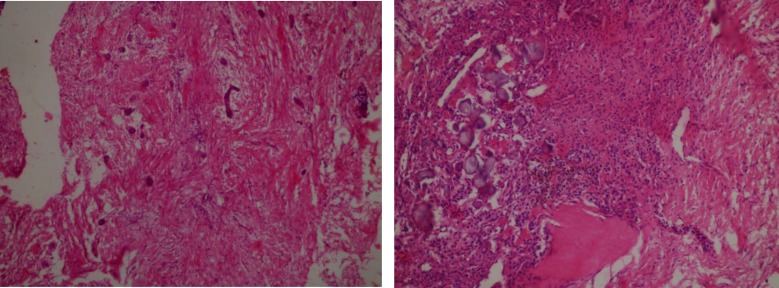
Photomicrograph showing fibrocellular CT stroma with strands of odontogenic epithelial islands composed of peripherally arranged tall columnar cells 10X (a); Deeper section showed the presence of few basophilic calcified areas 20X (b

On histopathological examination, the H & E section revealed a highly fibrocellular connective tissue stroma consisting of numerous strands of odontogenic epithelial islands composed of peripherally arranged tall columnar cells. The stroma consists of collagenous bundles with plump shaped fibroblasts and endothelial lined blood vessels with extravasated RBCs. Deeper section showed the presence of few basophilic calcified areas [Fig. 4 a, b]. Consider the clinical, radiological and histopathological features, final diagnosis of OF was given. The patient was kept under observation initially for 10 d and periodic follow-up was done for a year. No recurrence was reported.

## Discussion

COF represents about 9.4% of all odontogenic tumours. The most common odontogenic tumour is the odontoma with OF occupying the fourth highest position in order of tumour frequency (6). OF have a mesenchymal derivation of dental origin, that chiefly occurs before the fourth decade of life. A relative frequency of the tumour ranges between 0.1% and 6.1% among all other odontogenic tumours (7). Within the time period between 1954 to 2002, only 68 cases of odontogenic fibromas were found while recently, the data increased to 70 described cases until 2005 (8). In 1980, Gardner attempted to investigate and classified these lesions into three categories: 1) Hyperplastic dental follicle; 2) Simple type (fibrous neoplasm with collagenous fibrous connective tissue containing odontogenic epithelium); and 3) WHO type (lesion with dysplastic dentin or tissue like cementum and odontogenic epithelium) (6, 9).

Presently, WHO type (lesion with dysplastic hard matrix) should be termed as OF complex type or fibroblastic OF (9). Clinically, COF might begin as an asymptomatic expansion of the buccal or lingual cortical plates, taking place in the mandible and maxilla with equivalent occurrence. However, the lesion appears frequently to involve the anterior region, in the maxilla, whereas, in the mandible, it is likely to be located in the posterior area, involving the bicuspids and molar regions. A case of OF was reported in the maxillary right canine region (10). This feature was not in accordance with our case as the lesion was found in the posterior maxillary jaw region. This tumour appears in a wide age group [from 11 to 66 yr] with predilection for females [male: female ratio being 1:2.8] (2). The present reported case was found to be affecting an 18 yr old male patient.

Radiologically, it presents as uni- or multilocular radiolucency with distinct borders. In very few cases, it could present with a mixed radiolucent and radiopaque appearance ill-defined borders. Root resorption and displacement of teeth have been reported in cases of more severe lesions (9). Approximately, 12% of OF exhibit radiopaque flakes (4). The described lesion is at times coupled with an impacted or unerupted tooth. OF frequently exhibits an aggressive manner by invading the adjacent bone trabeculae and could pretend to give a malignant picture radiographically (7).

Microscopically, the lesion is characterized principally by dense mature collagen intermingled with fibroblasts and fibrocytes. The collagenous tissue being reasonably dense to dense in most areas and inactive odontogenic epithelium in strands or nests may be present (11). WHO central variant along with the same features of the simple type can also be characterized with odontogenic epithelium and calcifications analogous to cementum or dentine. Some of the other histopathological types comprise of granular cell type and hybrid tumour of central OF and giant cells. (5). Similar histological features were noticed in the present reported case.

As treatment, enucleation and curettage produce sufficiently favorable results. COF does not have a defined capsule, but it has limited potential for growth. The treatment is conventional surgery with minimal invasion through the enucleation of the neoplasm followed by the curettage of the cavity to enhance proper healing. Recurrences are not common. Among the cases reported in literature, few have shown recurrence. Thus, this pathology has a sufficiently favorable prognosis (5, 8).

## Conclusion

COF is an infrequent benign mesenchymal neoplasm with several subtypes originating due to several reasons that facilitate its pathogenesis and its subtypes. The importance of periodic clinical and radiographic examinations demonstrated for the timely detection of this kind of pathology. Moreover, it demonstrates the importance of adequate experience in the treatment and post-operative follow-up to detect the rare occurrence of the lesion.

## Conflict of Interests

The authors declare that there is no Conflict of Interests. 
